# Estimation of HIV incidence in Brazil, 2005-2011

**DOI:** 10.1186/1471-2334-14-S2-P23

**Published:** 2014-05-23

**Authors:** Celia Landmann Szwarcwald, Paulo Roberto Borges de Souza Júnior, Ana Roberta Pascom, Orlando Ferreira

**Affiliations:** 1 Fundação Oswaldo Cruz, Rio de Janeiro, Brazil

## Introduction

The incidence of HIV is nowadays the most valuable indicator of epidemiologic surveillance, both to guide prevention activities and to monitor ongoing interventions. This paper addresses a method to estimate the HIV incidence in Brazil in recent years.

## Methods

As the information source, we used the national lab information system (SISCEL) created to monitor CD4/CD8 count and viral load among HIV infected people. The proposed method is based on a statistical model that relates the first CD4 count to time of HIV seroconversion (Lodi et al., Clinical Infectious Diseases: 53(8): 817-825, 2011). To use the model in Brazil, we simulated the slope and the intercept for sex and age at first CD4 count. For each treatment-naïve HIV infected case reported in SISCEL aged 15 years and older, we estimated the time lag between HIV infection and the date the case was reported. The HIV incidence was estimated as the sum of the cases reported in the same year of infection, one year after infection, and so on up to 20 years after infection.

## Results

The average time from HIV infection to date of first CD4 count was 5 years. The proportion of cases reported to SISCEL in the same year of HIV infection increased from 30%, in 2005, to 36%, in 2011.The HIV incidence was estimated as 38968 (95% CI 36805-41130) in 2011, or 2.67 per 10000 population . The estimated HIV incidence represents approximately 5% of the HIV prevalence. The analysis by gender showed that the HIV incidence increased among men and decreased among women in the period 2005-2011. The male-female incidence ratio was 1.45 in 2005 but increased to 1.96 in 2011.

## Conclusions

In Brazil, where antiretroviral therapy is free and coverage is high, the survival of people living with HIV/AIDS is continuously increasing, and so is the HIV prevalence. Therefore, only studies that consider the HIV incidence can provide information on recent changes in transmission patterns. The results here depicted suggest that interventions in Brazil should be focused on young adult men.

